# DynEC: dynamic evolutionary clustering for power user load profiling using multi-view graph neural networks

**DOI:** 10.3389/frai.2026.1829649

**Published:** 2026-05-25

**Authors:** Lei Zhao, Hong Zhao, Mengjie Li, Jia Wang, Xingsi Ke, Yumin Yao

**Affiliations:** 1 State Grid Sichuan Electric Power Corporation, Chengdu, China; 2 School of Computer Science and Engineering, Central South University, Changsha, China

**Keywords:** concept drift, dynamic evolutionary clustering, graph neural networks, load profiling, multi-view learning, source-grid-load-storage

## Abstract

**Introduction:**

With the deep integration of generation-transmission-load-storage systems, the power demand side has become highly dynamic and stochastic, challenging the traditional assumption that user behavior remains stationary over time. Static clustering models therefore suffer from sensitivity to daily noise and false user identity switching.

**Methods:**

This study proposes Dynamic Evolutionary Clustering (DynEC), a multi-view graph neural network framework for power user load profiling. DynEC constructs a sparse multi-view dynamic graph that captures geometric proximity, temporal alignment through constrained dynamic time warping, and statistical dependencies. A gated spatiotemporal graph neural network then optimizes a dual-objective loss to learn latent representations while balancing current snapshot quality and historical temporal smoothness.

**Results:**

Experiments on real-world datasets show that DynEC outperforms existing baseline methods. The proposed framework identifies genuine concept drift more accurately while reducing erroneous cluster switching.

**Discussion:**

DynEC provides a stable and reliable profiling tool for modern power grid management by modeling load profiling as a continuous evolutionary process rather than a set of independent static clustering tasks.

## 1 Introduction

With the development of Advanced Metering Infrastructure (AMI) and artificial intelligence (Balamurugan et al., 2025; Belge et al., 2024), load forecasting in power grids has shifted from a passive interface to an active, bidirectional engagement point. This structural shift toward a “generation-grid-load-storage” paradigm necessitates a transition from macroscopic demand management to precise, user-centric profiling. At the core of this transformation lies load profiling, as precise load profiles serve as the foundation for dynamic pricing design, demand response (DR) targeting (Zou et al., 2024; Tolas et al., 2024; Zhang et al., 2025), and grid flexibility planning under carbon neutrality constraints (Badhe et al., 2025; Verma and Rao, 2025).

With the proliferation of distributed energy resources and electric vehicles, users are increasingly becoming grid prosumers who both consume and generate electricity. Consequently, influenced by real-time pricing, extreme weather events, and evolving work-from-home practices, daily consumption patterns have become highly stochastic. These factors continuously reshape the load curve, triggering concept drift (Gama et al., 2014; Lu et al., 2018). The traditional assumption that user behavior remains strictly stationary over time is no longer suitable for this pattern.

Current methods treat load profiling as a static snapshot problem, aggregating data from several months to assign users a single, permanent label. While useful for high-level infrastructure planning, static clustering fails severely in grids with a high proportion of renewable energy. When users fundamentally alter their routines (e.g., by purchasing an electric vehicle), static models exhibit delayed adaptation, leading to persistent misclassification. Conversely, if operators repeatedly apply static clustering to daily or weekly data blocks, the model overreacts to normal daily noise. Users may jump erratically between clusters without any actual underlying behavioral change. This false “identity switching” undermines the temporal smoothness required for reliable demand response (DR) targeting. Dynamic evolutionary clustering offers a solution by modeling profiles not as isolated snapshots, but as continuous videos that explicitly track how behavior evolves, while penalizing unstable cluster hopping.

It is well known that Euclidean metrics are highly vulnerable to temporal phase shifts (Kim et al., 2025). Two users may have identical consumption patterns, but if one wakes up an hour later, the Euclidean distance will place them in completely different clusters. Dynamic Time Warping (DTW) (Berndt and Clifford, 1994; Keogh and Ratanamahatana, 2005) addresses this alignment issue but suffers from poor scalability on large-scale datasets. Meanwhile, while spatiotemporal graph neural networks (ST-GNNs) have achieved some success in load forecasting, they lack the specific loss functions required to balance structural feature learning with temporal clustering consistency in unsupervised settings.

In summary, to bridge the gap between structural graph learning and dynamic pattern evolution, this study proposes DynEC (Dynamic Evolutionary Clustering). Our approach makes three main contributions: First, it constructs a multi-view dynamic graph to address the temporal evolution of user profiles. By integrating geometric proximity, temporal alignment (via computationally efficient cDTW), and statistical dependencies (Pearson correlation) into a unified graph structure, it reliably captures user relationships even under severe phase shifts. Second, we design a self-supervised deep evolutionary graph learning framework that combines a gated spatio-temporal graph encoder with a dual-objective evolutionary optimization strategy. By adopting a network-optimized dual loss function that balances snapshot quality and temporal smoothness, the model adapts to genuine concept drift while suppressing false identity switching. Third, we evaluate the framework using real-world smart meter data from three different cities. The results confirm that it achieves excellent internal clustering quality, as measured by the silhouette coefficient and ARI, while significantly reducing the cluster switching rate (CSR). By effectively tracking genuine behavioral shifts while ignoring daily noise, DynEC provides a highly stable and actionable profiling engine for next-generation smart grids.

The remainder of this article is structured as follows: Section 2 reviews the relevant literature. Section 3 provides a detailed description of the DynEC methodology and complexity analysis. Section 4 presents the experimental setup and results. Section 5 concludes the article.

## 2 Related work

### 2.1 Dynamic community detection and evolutionary clustering

The effective organization of dynamic data streams is a focal point of academic research. This field is typically divided into two main areas: dynamic community detection in social networks and evolutionary clustering in data mining. In particular, (Chakrabarti et al., 2006) proposed a formalized “evolutionary clustering” framework, noting that the optimal clustering solution at time *t* requires balancing two competing objectives: maximizing the current data fit (Snapshot Quality) while minimizing the deviation from previous clustering results (Temporal Cost). However, in traditional methods, the trade-off parameters are typically user-defined. To overcome this limitation, deep graph learning can automatically learn adaptive, dynamically evolving parameters.

Building on the perspectives proposed by Wang et al. (2019) and Tariq et al. (2022), this study adapts static clustering models for dynamic environments; Table 1 summarizes the conceptual differences between the static and dynamic paradigms.

**Table 1:** Comparison between static and dynamic clustering paradigms.

Dimension	Static clustering	Dynamic clustering
Metaphor	Static photo	Continuous video
Temporal perspective	Ignores time or flattens it (snapshot)	Explicitly models evolution
Feature representation	Global/static statistics	Time-varying embeddings
Primary goal	Discover long-term stable patterns	Capture pattern evolution and drift
Drift sensitivity	Cannot detect concept drift	Adaptable to and detects drift
Computational cost	Low (one-time)	High (online/recursive)
Application	Long-term planning	Real-time DR, anomaly detection

In the context of smart grids, user behavior often undergoes concept drift, causing the statistical properties of target variables to change over time (Gama et al., 2014; Jiang et al., 2021). Traditional static methods, such as applying K-Means to daily snapshots, typically overreact to noise. This can trigger “cluster hopping,” where users oscillate between clusters without any actual change in behavior (Jain et al., 2021). Conversely, incremental learning methods that update only cluster centroids suffer from “lag,” failing to adapt quickly to sudden shifts. Therefore, the framework proposed in this study adopts explicit modeling of nodes, incorporates temporal evolution, and updates cluster centroids, enabling the model to adapt to dynamic changes in clustering.

Recent advances in graph-stream mining have introduced methods that maintain temporal summaries of graph structures. For example, Time2Graph (Cheng et al., 2020) treats temporal evolution as a sequence of shapelets, but it still relies on static K-Means in the final clustering step. DynEC enhances existing methods by directly integrating temporal consistency into deep learning objectives.

### 2.2 Deep learning in load profiling

Current deep learning technologies significantly advance smart grid management by enabling robust load profile clustering and predictive pattern recognition. For instance, Long et al. (2025) combined density-based clustering (DBSCAN) with a graph attention network to predict air conditioning loads across geographic grids. This approach demonstrates the value of grouping spatially correlated units. In the context of data security, Zhang et al. (2025) proposed a CK-Means clustering scheme based on adaptive differential privacy for smart meter data analysis. This ensures both privacy protection and computational efficiency. Furthermore, Muyulema-Masaquiza and Ayala-Chauvin (2025) showed that effective consumption segmentation directly supports dynamic pricing, anomaly detection, and demand forecasting.

#### 2.2.1 Static deep clustering

Early studies typically employed dimension reduction techniques, such as principal component analysis (PCA) and hand-engineered features, followed by K-Means clustering (Chicco et al., 2006). Later, deep clustering methods based on autoencoders enabled the concurrent optimization of feature learning and cluster assignment. However, these methods are inherently static. They treat data as a collection of independent and identically distributed (i.i.d.) samples, ignoring the sequential dependencies inherent in load profiles. While effective for static snapshots, they fail to capture the temporal sequence in dynamic grid data. Therefore, adaptive modeling is crucial; Wang et al. (2020) specifically adopted deep reinforcement learning for demand response management.

#### 2.2.2 Spatio-temporal graph neural networks

To effectively capture complex spatial structures and temporal dependencies, advanced power grid analysis techniques typically employ spatio-temporal graph neural networks (ST-GNNs). Typical model architectures, such as Graph WaveNet (Wu et al., 2019) and DCRNN (Li et al., 2018), integrate graph convolutional networks (GCNs) with spatiotemporal convolutional or recurrent neural networks (RNNs). This integration simultaneously models spatial dependencies (power grid topology) and temporal dynamics (load trends). EvolveGCN (Pareja et al., 2020) uses RNNs to evolve the parameters of the GCN itself to adapt to changes in global distributions. However, for load profiling, preserving individual user identities is crucial. Therefore, TGN (Rossi et al., 2020) captures continuous temporal dynamics through a memory module to extract individual user profiling features. DySAT (Sankar et al., 2020) constructs adaptive dynamic profiles by introducing a self-attention mechanism to jointly model structural and temporal evolution. Furthermore, deep graph clustering has emerged as a highly promising direction. Works such as SDCN (Bo et al., 2020) have successfully integrated structural information into clustering tasks. Concurrently, contrastive learning methods (e.g., temporal subgraph contrastive learning Wang et al., 2023) have demonstrated potential in dynamic graph representations. This study focuses on evolving node embeddings via GRU memory modules to ensure the model retains stable historical context for each specific user, thereby preventing identity loss during temporal updates.

Despite significant progress in the aforementioned areas, most spatiotemporal graph neural network (ST-GNN) research has primarily focused on supervised learning tasks, such as load estimation (Fekri et al., 2021) or fault detection. Consequently, the exploration of unsupervised clustering remains very limited. Foundational work, such as DGI (Veličković et al., 2019) and MVGRL (Hassani and Khasahmadi, 2020), has demonstrated the feasibility of unsupervised graph representation learning. However, applying these principles to power grid user profiling with dynamically evolving features remains challenging.

### 2.3 Similarity measures: geometric, shape-based, and statistical

In user profiling tasks based on Graph Neural Networks (GNNs), constructing meaningful user graphs is critical to model performance. Although the commonly used Euclidean distance offers an ideal computational complexity of O(D), it is highly sensitive to temporal phase shifts when processing time-series data like load curves. Even minor phase shifts can incorrectly separate inherently similar curves, a behavior demonstrated in experimental analyses by Kim et al. (2025).

To address this issue, we incorporate Dynamic Time Warping (DTW). As emphasized by Berndt and Clifford (1994), DTW effectively compensates for local deviations along the time axis by finding an optimal non-linear alignment. However, standard DTW has a time complexity of $O\left(D^{2}\right)$, making it computationally expensive for large-scale applications. For load curves with prominent periodicity, Constrained Dynamic Time Warping (cDTW) offers a more efficient alternative. The pioneering work of Sakoe and Chiba (1978) demonstrates that introducing Sakoe–Chiba bands to apply local constraints on the alignment path maintains accuracy while controlling time complexity within a polynomial range of the number of data points *D* and the signal-to-noise ratio *w*. A more systematic review can be found in Ratanamahatana and Keogh (2004).

Building on this, recent studies have also explored mapping DTW similarity into Euclidean space via Shapelets to explicitly enforce alignment constraints during synthesis (El Amouri et al., 2023). However, the automatic Shapelet discovery process often requires intensive computation, causing practical difficulties in large-scale situations.

To combine the strengths of various similarity measures, Lin et al. (2019) proposed integrating multiple metrics. This strategy incorporates geometric similarity (e.g., Euclidean distance), shape similarity (cDTW), and statistical dependence (specifically, the Pearson correlation coefficient in our work) into a multidimensional graph, thereby enabling the construction of more robust graph structures in the presence of noise.

### 2.4 Clustering validation and stability analysis

Traditional clustering validity metrics, such as the Rousseeuw coefficient (Rousseeuw, 1987) or the Davies–Bouldin index, primarily focus on snapshot quality, verifying the compactness and separation of clusters. They ignore temporal instability and impose no penalty when users jump erratically between clusters.

As noted by Jain et al. (2021), in industrial applications, stability is just as important as accuracy. A clustering algorithm that produces drastically different partitions when the input data is slightly perturbed is operationally useless. In dynamic settings, this translates directly to temporal stability: unless the underlying data distribution actually shifts, the cluster assignments should not change significantly between time steps (Lange et al., 2004; Luxburg, 2007). We use the cluster switching rate (CSR) and temporal smoothness (TS) metrics to formalize this requirement. This dual-metric system provides a holistic evaluation framework that balances internal cluster quality with operational robustness.

## 3 Methodology

### 3.1 Problem definition

This study formalizes the dynamic user segmentation problem under concept drift as a dynamic graph clustering task. Rather than treating electricity consumers as isolated entities with static labels, we model the continuous evolution of their energy consumption behaviors through complex spatio-temporal interactions. By representing consumers as nodes and their multi-view similarities as time-varying edges, the challenge of tracking genuine behavioral shifts is mathematically transformed into an evolutionary representation learning and clustering optimization problem over a sequence of dynamic graphs.

#### 3.1.1 Prerequisites

**Definition 1** (Dynamic load profile stream (DLPS)). Let *U* = {*u*_1_, *u*_2_, …, *u*_*N*_} be the set of electricity users, where *N* is the total number of users. At each time step *t* ∈ {1, 2, …, *T*}, we observe a load matrix *X*^(*t*)^. Let $x_{i}^{\left(t\right)}\in {\mathbb{R}}^{D}$ denote the electricity consumption profile of user *u*_*i*_ over a time interval of length *D*. The continuous load profile stream is thus defined as Equation 1:


$$\begin{array}{l} X=\left\{X^{\left(1\right)},X^{\left(2\right)},\ldots ,X^{\left(T\right)}\right\} \end{array}$$ (1)

**Definition 2** (Concept drift). A data stream is considered to exhibit concept drift when its underlying joint probability distribution changes over time, as shown in Equation 2:


$$\begin{array}{l} P\left(X^{\left(t\right)}\right)\neq P\left(X^{\left(t+1\right)}\right) \end{array}$$ (2)

In the context of power grids, such drift typically arises from individual behavioral shifts, such as installing new appliances or developing new electric vehicle charging patterns, or from system-level macro changes, such as seasonal effects or pricing adjustments.

**Definition 3** (Dynamic graph flow). We model the evolving relationships among users as a sequence of dynamic graphs, as shown in Equation 3:


$$\begin{array}{l} G=\left\{G^{\left(1\right)},G^{\left(2\right)},\ldots ,G^{\left(T\right)}\right\} \end{array}$$ (3)

Here, the node set *V* = *U* remains fixed, while the time-varying adjacency matrix *A*^(*t*)^ captures the instantaneous similarities between users at time *t*.

#### 3.1.2 Problem description

Given a sequence of dynamic graphs G, our objective is to learn a mapping function *f*_θ_ at each time step *t*, as shown in Equation 4:


$$\begin{array}{l} f_{\theta }:G^{\left(t\right)}\to C^{\left(t\right)} \end{array}$$ (4)

where $C^{\left(t\right)}=\left\{C_{1},\ldots ,C_{K}\right\}$ denotes the cluster partition of the *N* users into *K* distinct groups. This partition must simultaneously optimize two competing objectives:

**Snapshot quality:** Users within the same cluster *C*_*k*_ should exhibit high intra-cluster similarity in terms of geometric characteristics (e.g., load magnitude and shape), temporal synchronization, and statistical dependencies.

**Temporal consistency:** The clustering structure should evolve smoothly over time. Specifically, the current partition $C^{\left(t\right)}$ should not deviate drastically from the previous partition $C^{\left(t-1\right)}$ unless genuine concept drift occurs, thereby minimizing erroneous user identity switching.

### 3.2 Framework architecture

**Figure 1:**
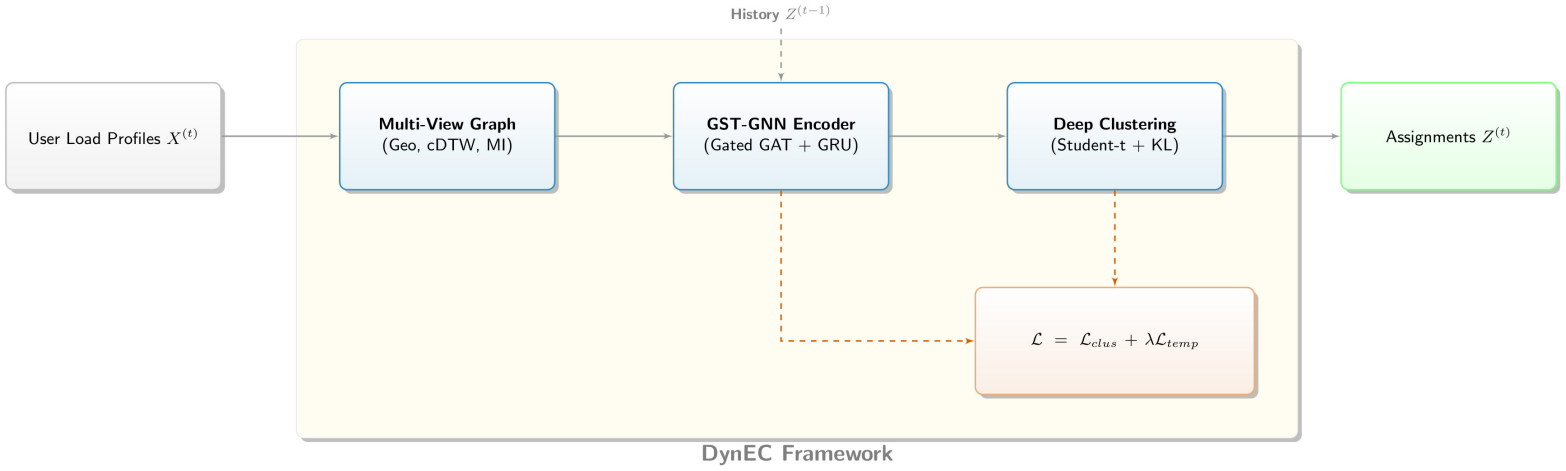
Overview of the architecture of DynEC. **(A)** Multi-view dynamic graph construction: fusing geometric, temporal, and statistical dependencies. **(B)** Gated spatio-temporal graph encoder: updating node embeddings via Multi-Head GAT and GRU. **(C)** Dual-objective clustering: optimizing for both cluster purity (KL divergence to target) and temporal consistency.

As illustrated in Figure 1, the proposed Dynamic Evolutionary Clustering framework consists of two core modules that operate sequentially at each time step.

First, the **Multi-view dynamic graph construction** module (Section 3.3) takes the raw multi-dimensional load profiles as input and constructs a comprehensive user relationship graph. Rather than relying on a single similarity metric, it models inter-user relationships from three complementary perspectives: geometric proximity (Euclidean distance), temporal alignment (Constrained Dynamic Time Warping, cDTW), and statistical dependency (Pearson correlation). This multi-view fusion mechanism is specifically designed to capture complex non-linear correlations and robustly align user behaviors even in the presence of severe temporal phase shifts.

Second, the **Deep evolutionary graph learning** module (Section 3.4) performs evolutionary representation learning and clustering assignment. It integrates a Gated Spatio-Temporal Graph Encoder (comprising a Multi-Head Graph Attention Network and a Gated Recurrent Unit) to extract evolution-aware node embeddings. These embeddings are then optimized through a Dual-Objective Optimization mechanism that explicitly balances the snapshot clustering quality (via KL divergence) with temporal smoothness. By jointly optimizing these two objectives, the architecture effectively solves the stability-plasticity dilemma, enabling the reliable tracking of genuine concept drift while aggressively suppressing erroneous identity switching caused by daily noise.

### 3.3 Multi-view dynamic graph construction

This section elucidates the rationale behind the multi-view framework, detailing the specific relationships characterized by each view and the mechanism for fusing them into a unified graph structure. We construct the user similarity structure from three complementary perspectives—geometric proximity, temporal alignment, and statistical dependency—to facilitate robust graph representation learning.

#### 3.3.1 Motivation: toward a comprehensive user relationship graph

At each time step *t*, the objective is to construct a dynamic graph *G*^(*t*)^ = (*V, E*^(*t*)^) that authentically reflects the multifaceted relationships among users. A single metric, such as Euclidean distance, is often insufficient as it primarily captures similarity in *numerical magnitude at synchronized sampling points*. Consequently, it fails to account for: load profiles that exhibit similar morphological patterns but are subject to temporal phase shifts, as well as users who display significant disparities in magnitude or phase but still share underlying statistical dependencies. To address these limitations, we adopt a multi-view modeling approach, synthesizing three distinct adjacency structures to derive a unified adjacency matrix *A*^(*t*)^.

#### 3.3.2 Geometric view (*A*_*geo*_): characterizing magnitude-based proximity

The geometric view captures the local proximity of user load profiles within the Euclidean space at a given time grid. For any pair of users (*i, j*), the Euclidean distance at time *t* is defined as Equation 5:


$$\begin{array}{l} d_{geo}\left(i,j\right)=||x_{i}^{\left(t\right)}-x_{j}^{\left(t\right)}{||}_{2}. \end{array}$$ (5)

Based on this metric, a k-Nearest Neighbors (kNN) graph is constructed: *A*_*geo*_(*i, j*) = 1 if user *j* is among the *k*-nearest neighbors of user *i*, and 0 otherwise. This view connects users exhibiting *highly synchronized consumption variations* predominantly on the same temporal scale, thereby reflecting local geometric similarities.

#### 3.3.3 Temporal alignment view (*A*_*dtw*_): characterizing shape similarity under temporal shifts

A critical limitation of the geometric view is its sensitivity to temporal phase shifts. For instance, two users with morphologically similar load curves may be deemed dissimilar by Euclidean distance due to a time lag (e.g., cooking activities occurring at different times). To mitigate this, the temporal alignment view incorporates *Constrained Dynamic Time Warping* (cDTW). cDTW minimizes the cumulative distance between matched points by identifying an optimal warping path *W* = {*w*_1_, …, *w*_*K*_} between two sequences *x*_*i*_ and *x*_*j*_ as shown in Equation 6:


$$\begin{array}{l} \text{cDTW}\left(x_{i},x_{j}\right)=\underset{W}{\min}\overset{K}{\underset{k=1}{\sum }}w_{k}, \end{array}$$ (6)

subject to the constraint |*i*_*k*_ − *j*_*k*_| < *w*, where *w*_*k*_ denotes the distance of the *k*-th matched pair and *w* is the Sakoe–Chiba window width. Given the inherent periodicity of daily load profiles, imposing a narrow window *w* reduces the time complexity to O(D·w), ensuring approximately linear scalability while preserving alignment precision.

Upon computing the cDTW distance, a Gaussian kernel is employed to transform the distance into a similarity measure as shown in Equation 7:


$$\begin{array}{l} S_{dtw}\left(i,j\right)=\exp\left(-d^{2}\left(i,j\right)/\sigma^{2}\right). \end{array}$$ (7)

The resulting similarity matrix is subsequently thresholded to yield a sparse adjacency matrix *A*_*dtw*_. This view effectively links users whose *load profiles are morphologically similar but temporally shifted*, serving as a crucial complement to the geometric view.

#### 3.3.4 Correlation view (*A*_*corr*_): characterizing statistical dependency

Two load profiles may differ significantly in magnitude or phase yet still exhibit strong co-movement patterns. The dependency view, therefore, uses the absolute Pearson correlation coefficient to quantify statistical dependency between users while remaining computationally efficient for high-dimensional continuous load profiles. For two load profiles *x*_*i*_ and *x*_*j*_, the correlation score is defined as Equation 8:


$$\begin{array}{l} \rho \left(i,j\right)=\left|\frac{{\left(x_{i}-{\bar{x}}_{i}\right)}^{⊤}\left(x_{j}-{\bar{x}}_{j}\right)}{||x_{i}-{\bar{x}}_{i}{||}_{2}||x_{j}-{\bar{x}}_{j}{||}_{2}}\right|. \end{array}$$ (8)

This metric facilitates the identification of users with statistically similar behavior even in the presence of *substantial discrepancies in the raw time domain*. The corresponding adjacency matrix *A*_*corr*_ therefore captures *linear statistical dependency*, enriching the graph with information complementary to geometric proximity and temporal alignment.

#### 3.3.5 Graph fusion: integration of heterogeneous views

The three views generate distinct adjacency matrices $A_{geo}^{\left(t\right)}$, $A_{dtw}^{\left(t\right)}$, and $A_{corr}^{\left(t\right)}$. To enable the Graph Neural Network to learn from these heterogeneous sources, we employ a “weighted fusion and normalization” strategy to synthesize a unified adjacency matrix *A*^(*t*)^.

First, the views are integrated via a weighted linear combination as shown in Equation 9:


$$\begin{array}{l} A_{fused}^{\left(t\right)}=\alpha A_{geo}^{\left(t\right)}+\beta A_{dtw}^{\left(t\right)}+\gamma A_{corr}^{\left(t\right)}, \end{array}$$ (9)

where α, β, γ are hyperparameters satisfying α+β+γ = 1, regulating the relative contributions of geometric proximity, temporal alignment, and statistical dependency.

Subsequently, symmetric normalization is applied to the fused adjacency matrix $A_{fused}^{\left(t\right)}$ as shown in Equation 10:


$$\begin{array}{l} A^{\left(t\right)}=D^{-\frac{1}{2}}\left(A_{fused}^{\left(t\right)}+I\right)D^{-\frac{1}{2}}, \end{array}$$ (10)

where *I* is the identity matrix representing self-loops, and *D* is the degree matrix of $A_{fused}^{\left(t\right)}+I$. This normalization is essential for mitigating the bias introduced by uneven degree distributions during graph convolution.

The resulting unified adjacency matrix *A*^(*t*)^ simultaneously encodes local Euclidean proximity (Geometric View), shape similarity invariant to temporal shifts (Temporal Alignment View), and linear statistical dependency (Correlation View). This multi-view dynamic graph provides a comprehensive and robust structural foundation for subsequent spatio-temporal learning in the GST-GNN.

The detailed procedure for multi-view dynamic graph construction is summarized in Algorithm 1.

**Algorithm 1:** Multi-view dynamic graph construction.

**Require:** Load profiles *X*^(*t*)^, Neighbors *k*, Window *w*, Kernel σ, Weights α, β, γ.
**Ensure:** Fused Adjacency Matrix *A*^(*t*)^.
1: **Geometric View Construction:**
2: Compute pairwise Euclidean distances *D*_*geo*_;
3: Construct kNN graph $A_{geo}^{\left(t\right)}$ based on *D*_*geo*_ with *k* neighbors;
4: **Temporal Alignment View Construction:**
5: Compute pairwise cDTW distances *D*_*dtw*_ with window *w* (Equation 5);
6: Convert to similarity $S_{dtw}=\exp\left(-D_{dtw}^{2}/\sigma^{2}\right)$;
7: Sparsify *S*_*dtw*_ to obtain $A_{dtw}^{\left(t\right)}$;
8: **Dependency View Construction:**
9: Compute Pearson-correlation matrix $A_{corr}^{\left(t\right)}$ (Equation 6);
10: **Fusion and Normalization:**
11: $A_{fused}^{\left(t\right)}\leftarrow \alpha A_{geo}^{\left(t\right)}+\beta A_{dtw}^{\left(t\right)}+\gamma A_{corr}^{\left(t\right)}$;
12: Normalize $A^{\left(t\right)}\leftarrow D^{-\frac{1}{2}}\left(A_{fused}^{\left(t\right)}+I\right)D^{-\frac{1}{2}}$;
13: **return** *A*^(*t*)^

### 3.4 Deep evolutionary graph learning framework

The core of DynEC is a unified self-supervised learning framework that seamlessly integrates a Gated Spatio-Temporal Graph Encoder with a Dual-Objective Optimization Mechanism.

#### 3.4.1 Gated spatio-temporal graph encoder

The Gated Spatio-Temporal Graph Neural Network (GST-GNN) is designed to learn node embeddings that capture both the structural patterns from the multi-view graph *A*^(*t*)^ and the temporal evolution of user behaviors from the feature stream *X*^(*t*)^. Unlike traditional ST-GNNs, which primarily focus on supervised forecasting tasks (Wu et al., 2019), our encoder is specifically tailored for *unsupervised evolutionary clustering*, effectively connecting static structural learning with dynamic pattern evolution. The “Gated” nature of this architecture is twofold: it employs an attention-based soft gating mechanism for spatial aggregation and a gated recurrent unit for temporal updates.

##### 3.4.1.1 Spatial aggregation (multi-head GAT)

To capture the manifold relationship patterns present in the multi-view graph, a Multi-Head Graph Attention Network (GAT) is employed. Let $h_{i}^{\left(t\right)}$ denote the feature vector of node *i* at time *t* (where initially $h_{i}^{\left(t\right)}=x_{i}^{\left(t\right)}$). For each attention head *k*, the attention coefficient *e*_*ij, k*_ between node *i* and its neighbor $j\in N_{i}$ (defined by *A*^(*t*)^) is calculated as follows Equation 11:


$$\begin{array}{l} e_{ij,k}=\text{LeakyReLU}\left({\vec{a}}_{k}^{T}\left[W_{k}h_{i}^{\left(t\right)}||W_{k}h_{j}^{\left(t\right)}\right]\right) \end{array}$$ (11)

where *W*_*k*_ denotes the learnable weight matrix, ${\vec{a}}_{k}$ is the attention vector for the *k*-th head, and || represents the concatenation operation. The attention weights, denoted by α_*ij, k*_, are obtained through softmax normalization as shown in Equation 12:


$$\begin{array}{l} \alpha_{ij,k}=\frac{\exp\left(e_{ij,k}\right)}{\sum_{l\in N_{i}}\exp\left(e_{il,k}\right)} \end{array}$$ (12)

These weights function as a “soft gating” mechanism, filtering out noisy connections by assigning lower importance to irrelevant neighbors. The final spatial embedding $h_{i,\text{spat}}^{\left(t\right)}$ is obtained by concatenating the outputs of the *K* heads as shown in Equation 13:


$$\begin{array}{l} h_{i,\text{spat}}^{\left(t\right)}=\overset{K}{\underset{k=1}{||}}\sigma \left(\underset{j\in N_{i}}{\sum }\alpha_{ij,k}W_{k}h_{j}^{\left(t\right)}\right) \end{array}$$ (13)

where σ is a non-linear activation function (e.g., ELU).

##### 3.4.1.2 Temporal evolution (GRU update)

To capture user profile dynamics and handle concept drift, a Gated Recurrent Unit (GRU) is utilized to update the node embeddings. The GRU efficiently tracks temporal embeddings, providing a robust mechanism to update user representations under concept drift. The GRU processes the current spatial feature $h_{i,\text{spat}}^{\left(t\right)}$ (output from GAT) and the previous user embedding $z_{i}^{\left(t-1\right)}$ as input to yield the final temporal embedding $z_{i}^{\left(t\right)}$. The reset gate within the GRU determines the extent to which past information should be disregarded, a process essential for adapting to concept drift. Meanwhile, the update gate controls the incorporation of new spatial information. This mechanism enables the model to retain a long-term memory of user identity while adapting to short-term fluctuations.

#### 3.4.2 Dual-objective evolutionary optimization

The fundamental principle of our framework is unsupervised clustering. We employ a self-training methodology utilizing a Student's t-distribution kernel.

##### 3.4.2.1 Soft assignment (student's t-distribution)

The probability of user *i* belonging to cluster *k*, denoted *q*_*ik*_, is measured by the similarity between its embedding *z*_*i*_ and the cluster centroid μ_*k*_. In this study, we propose using the Student's t-distribution kernel instead of the conventional Gaussian kernel used in Gaussian Mixture Models. This choice is motivated by the heavy-tailed property of the t-distribution, which makes the clustering more robust to outliers. This is a critical feature when dealing with volatile electricity load data, where spikes and anomalies are common. The soft assignment is computed as follows Equation 14:


$$\begin{array}{l} q_{ik}=\frac{{\left(1+||z_{i}-\mu_{k}{||}^{2}/\nu \right)}^{-\frac{\nu +1}{2}}}{\sum_{k^{\prime }}{\left(1+||z_{i}-\mu_{k^{\prime }}{||}^{2}/\nu \right)}^{-\frac{\nu +1}{2}}} \end{array}$$ (14)

where ν represents the degrees of freedom, which is set to 1 in this instance, reducing the equation to a Cauchy distribution. This formulation enables a more flexible cluster boundary, accommodating the inherent noise in smart meter data without excessively penalizing distant points.

##### 3.4.2.2 Self-training target distribution

To overcome the lack of ground truth labels in unsupervised settings, we employ a self-training strategy. The target distribution, defined herein as *P*, is used to “sharpen” the soft assignments, *Q*, to encourage high-confidence predictions. The target probability *p* is derived from *q* by raising it to the second power and normalizing by cluster frequency as shown in Equation 15:


$$\begin{array}{l} p_{ik}=\frac{q_{ik}^{2}/f_{k}}{\sum_{k^{\prime }}q_{ik^{\prime }}^{2}/f_{k^{\prime }}} \end{array}$$ (15)

where $f_{k}=\underset{i}{\sum }q_{ik}$ is the soft cluster frequency. The target distribution is designed to satisfy three key properties: (1) Sharpening: By squaring the probabilities, the distribution is pushed toward a one-hot encoding, reducing entropy and forcing the model to make decisive cluster assignments. (2) Confidence Emphasis: Data points that initially demonstrate high confidence contribute more to the gradient, guiding the learning process. (3) Normalization: Division by *f*_*k*_ prevents large clusters from dominating the loss function, ensuring that smaller but distinct user groups (e.g., EV owners) are not ignored.

##### 3.4.2.3 Dual-objective loss function

The learning objective is divided into two competing components: clustering quality loss and temporal smoothness loss as shown in Equation 16.


$$\begin{array}{l} L=L_{clus}+\lambda L_{temp} \end{array}$$ (16)

**1. Snapshot clustering loss (**$L_{clus}$**):** This term minimizes the Kullback-Leibler (KL) divergence between the soft assignment *Q*^(*t*)^ and the target distribution *P*^(*t*)^ at the current time step as shown in Equation 17.


$$\begin{array}{l} L_{clus}=KL\left(P^{\left(t\right)}||Q^{\left(t\right)}\right)=\underset{i}{\sum }\underset{k}{\sum }p_{ik}^{\left(t\right)}\log\frac{p_{ik}^{\left(t\right)}}{q_{ik}^{\left(t\right)}} \end{array}$$ (17)

By minimizing this divergence, the model is forced to iteratively refine its cluster assignments, moving the centroids toward the high-density centers of the embeddings.

**2. Temporal consistency loss (**$L_{temp}$**):** To prevent erratic “Identity Switching,” we introduce a temporal smoothness constraint. This term penalizes abrupt deviations of the current cluster assignment *Q*^(*t*)^ from the previous assignment *Q*^(*t*−1)^ as shown in Equation 18.


$$\begin{array}{l} L_{temp}=KL\left(Q^{\left(t-1\right)}||Q^{\left(t\right)}\right)=\underset{i}{\sum }\underset{k}{\sum }q_{ik}^{\left(t-1\right)}\log\frac{q_{ik}^{\left(t-1\right)}}{q_{ik}^{\left(t\right)}} \end{array}$$ (18)

This regularization encourages the model to preserve a user's cluster membership unless strong evidence from new data dictates a change. The hyperparameter λ controls the trade-off between fitting the current snapshot (Plasticity) and respecting historical consistency (Stability).

#### 3.4.3 Overall training process

To provide a clear roadmap of the proposed methodology, the complete execution pipeline of the evolutionary clustering framework is summarized in Algorithm 2. The procedure operates in an online manner across continuous time steps. It begins with a pre-training phase to establish robust initial representations and cluster centroids. Subsequently, at each time step *t*, the framework sequentially performs multi-view graph construction, spatio-temporal embedding updates via the GST-GNN, and dual-objective optimization. This iterative process ensures that the model dynamically adapts to emerging concept drifts while preserving the structural continuity of user profiles.

**Algorithm 2:** Evolutionary clustering training process.

**Require:** Continuous load profile stream $X=\left\{X^{\left(1\right)},\ldots ,X^{\left(T\right)}\right\}$, Number of clusters *K*, Hyperparameters λ, α, β, γ.
**Ensure:** Cluster assignments $C=\left\{C^{\left(1\right)},\ldots ,C^{\left(T\right)}\right\}$ for all users.
1: **Initialization:** Pre-train the GST-GNN encoder using reconstruction loss; Initialize cluster centroids μ^(0)^ via K-Means on the initial embeddings *Z*^(0)^.
2: **for** *t* = 1 to *T* **do**
3: **Multi-View Graph Construction:** Compute adjacency matrices $A_{geo}^{\left(t\right)},A_{dtw}^{\left(t\right)},A_{corr}^{\left(t\right)}$.
4: Fuse and normalize into a unified graph *A*^(*t*)^ (Equation 7).
5: **Spatio-Temporal Embedding Update:**
6: Extract spatial features: *H*^(*t*)^←GAT(*A*^(*t*)^, *X*^(*t*)^).
7: Update temporal states: *Z*^(*t*)^←GRU(*H*^(*t*)^, *Z*^(*t*−1)^).
8: **Clustering & Dual-Objective Optimization:**
9: Compute soft assignments *Q*^(*t*)^ using Eq. Equation 11.
10: Calculate the auxiliary target distribution *P*^(*t*)^ using Eq. Equation 12.
11: Calculate clustering loss $L_{clus}$ and temporal consistency loss $L_{temp}$.
12: Update network parameters θ and cluster centroids μ by minimizing $L=L_{clus}+\lambda L_{temp}$.
13: **Cluster Assignment:** Assign each user *i* to cluster $c_{i}^{\left(t\right)}\leftarrow \arg\underset{k}{\max}q_{ik}^{\left(t\right)}$.
14: **end for**
15: **return** C.

## 4 Experiments

### 4.1 Experimental setup

#### 4.1.1 Datasets

To evaluate the performance of DynEC, we utilized three real-world smart meter datasets collected from distinct cities in Sichuan Province, China, covering the entire year of 2024. City A (Mixed Residential/Commercial) comprises 800 users, including a significant proportion of early adopters of Distributed Energy Resources (DERs) and Electric Vehicles (EVs). This dataset exhibits high volatility and frequent pattern shifts. City B (Residential-Dominant) consists of 500 users who display regular weekly patterns, although these patterns are subject to significant seasonal fluctuations. City C (Industrial Park Zone) contains 650 users from a specialized industrial environment, serving as a proxy for a fully integrated Source-Grid-Load-Storage system characterized by diverse interaction patterns and distinct concept drifts.

##### 4.1.1.1 Pre-processing

The raw AMI data were sampled at 15-min intervals and subsequently aggregated into hourly load profiles (*D* = 24). To simulate a realistic dynamic environment, we employed a sliding window approach with a sequence length of 24 h and a step size of 1 h. Missing values (approximately 0.5%) were imputed using linear interpolation. To scale each user's daily profile to the interval [0, 1], we applied min-max normalization. This ensures that the clustering process focuses on shape patterns rather than absolute magnitudes.

##### 4.1.1.2 Baseline information

To assess DynEC, we employed a comprehensive suite of baselines, comprising both static and dynamic methods. Evol-KMeans denotes our implementation of the evolutionary K-Means approach proposed by Chakrabarti et al. (2006), instantiated via centroid smoothing across consecutive monthly snapshots. The specific descriptions of these baselines are shown in Table 2.

**Table 2:** Summary of baseline methods.

Category	Method	Description
Static baselines	K-Means	Standard baseline for load profiling; acts as a reference for snapshot quality but suffers from high instability.
	Spectral Clustering	Clusters via Laplacian eigendecomposition (Luxburg, 2007); it is computationally expensive and lacks temporal consistency.
Dynamic/evolutionary baselines	Time2Graph	Shapelet-based temporal representation model adapted for clustering using K-Means, following the dynamic shapelet framework of Cheng et al. (2020).
	EvolveGCN-Clus	Adaptation of EvolveGCN (Pareja et al., 2020) evolving GCN parameters via RNN, modified for unsupervised clustering.
	Evol-KMeans	Evolutionary K-Means baseline with explicit centroid smoothing between consecutive monthly snapshots; designed to reduce switching but prone to under-adaptation when heterogeneous drift occurs.

### 4.2 Implementation details

#### 4.2.1 Fusion weight strategy

While the conceptual framework allows for dynamically updated fusion weights, our practical engineering implementation utilizes fixed, equal weights (α = β = γ = 1/3). This choice prevents “view collapse” during training and ensures robust performance across different datasets. Sensitivity to this design choice is analyzed in Section 4.6.

The DynEC framework was implemented in PyTorch Geometric. The specific parameter settings are listed in Table 3.

**Table 3:** Detailed implementation specifications and hyperparameter configuration.

Parameter	Value/specification
Model architecture	
Implementation framework	PyTorch geometric (PyG)
Graph encoder	2-layer Gated-GAT (Hidden dimensions: 64 → 32)
Temporal module	GRU (Hidden dimension: 32)
Attention mechanism	Multi-head Attention (*K* = 4 heads)
15.6-7.2,-14242ptClustering kernel	Student's t-distribution (Degrees of freedom ν = 1)
Hyperparameters	
Temporal consistency (λ)	0.1 (Selected via grid search)
Fusion weights (α, β, γ)	Fixed at 1/3 (Equal Weighting)
cDTW bandwidth	2 (equivalent to ±2 hours temporal shift)
Optimization	Adam optimizer (learning rate η = 10^−3^, Weight decay 10^−4^)
Training & hardware	
Pre-training strategy	50 epochs (MSE reconstruction loss)
Clustering strategy	100 epochs (KL divergence loss)
Batch size	256 users
Computing resources	NVIDIA RTX 3090 GPU (24GB VRAM), 64GB RAM

##### 4.2.1.1 Hyperparameters

The temporal consistency weight λ was set to 0.1, determined through a grid search on the validation set. For the graph fusion weights, fixed equal values of 1/3 were used for α, β, and γ throughout training. For the cDTW calculation, the Sakoe–Chiba bandwidth was set to 2, allowing for a temporal shift of up to 2 h. We utilized the Adam optimizer with a learning rate of 10^−3^ and a weight decay of 10^−4^.

##### 4.2.1.2 Training strategy

The GST-GNN encoder was pre-trained for 50 epochs using Mean Squared Error (MSE) reconstruction loss to initialize the node embeddings. Subsequent clustering training was conducted in mini-batches of 256 users at each time step for 100 epochs. All experiments were performed on a server equipped with an NVIDIA RTX 3090 GPU and 64GB of RAM.

### 4.3 Evaluation metrics

To evaluate the trade-off between clustering quality and temporal stability, we adopted a dual-metric evaluation system.

#### 4.3.1 Snapshot quality metrics

These metrics evaluate the degree to which the clustering structure corresponds to the data at a given time *t*. The Silhouette Coefficient (SC), developed by Rousseeuw (1987), is used to measure the contrast between intra-cluster cohesion and inter-cluster separation. Additionally, the Davies-Bouldin Index (DBI) is employed to evaluate the average similarity of each cluster with its most similar neighbor. When groundtruth labels are available, the Adjusted Rand Index (ARI) is also reported to quantify the agreement between predicted partitions and the reference clustering. In this study, ARI was computed against the pre-defined base_cluster labels in users.csv. These reference labels were maintained in the utility's operational system and were cross-checked against customer-type records (e.g., residential and commercial categories). To improve label reliability, domain experts from the State Grid Sichuan Electric Power Corporation randomly sampled 10% of users and verified the authenticity of their assigned user types against field-visit records before model development. The de-identified dataset and reference labels are publicly available in a GitHub repository.

#### 4.3.2 Temporal stability metrics

In addition to static quality, assessing the temporal consistency of cluster assignments is crucial for dynamic profiling. The Cluster Switching Rate (CSR) is used to measure the stability of user allocation over time. CSR is defined as the average proportion of users who change their cluster affiliation between consecutive time steps as shown in Equation 19:


$$\begin{array}{l} \text{CSR}=\frac{1}{T-1}\overset{T-1}{\underset{t=1}{\sum }}\frac{\sum_{i=1}^{N}1\left(c_{i}^{\left(t\right)}\neq c_{i}^{\left(t+1\right)}\right)}{N} \end{array}$$ (19)

where **1**(·) is the indicator function, and $c_{i}^{\left(t\right)}$ represents the cluster label of user *i* at time *t*. A lower CSR indicates higher temporal stability, meaning the model is robust to minor fluctuations and can capture consistent behavioral patterns.

### 4.4 Comparative analysis

As shown in Table 4, this study presents a comprehensive performance comparison with five baseline models across three cities. The results indicate that the framework proposed in this study achieves certain improvements in balancing clustering quality and temporal stability.

**Table 4:** Performance comparison on three datasets.

Method	City A (mixed)				City B (residential)				City C (industrial)			
	ARI ↑	SC ↑	DBI ↓	CSR ↓	ARI ↑	SC ↑	DBI ↓	CSR ↓	ARI ↑	SC ↑	DBI ↓	CSR ↓
K-Means	0.46 ± 0.00	0.41 ± 0.00	0.94 ± 0.00	0.80 ± 0.04	0.91 ± 0.00	0.59 ± 0.00	0.56 ± 0.00	0.70 ± 0.10	0.56 ± 0.00	0.45 ± 0.00	0.79 ± 0.00	0.78 ± 0.09
Spectral	0.51 ± 0.01	0.33 ± 0.01	1.09 ± 0.03	0.79 ± 0.06	0.51 ± 0.04	0.19 ± 0.04	1.26 ± 0.09	0.84 ± 0.04	0.47 ± 0.01	0.27 ± 0.02	1.11 ± 0.06	0.79 ± 0.05
EvolveGCN-Clus	0.44 ± 0.03	0.39 ± 0.02	1.01 ± 0.07	0.61 ± 0.04	0.90 ± 0.02	0.58 ± 0.01	0.59 ± 0.01	0.29 ± 0.10	0.62 ± 0.07	0.40 ± 0.01	0.96 ± 0.04	0.56 ± 0.04
Time2Graph	0.18 ± 0.01	0.17 ± 0.01	3.54 ± 0.41	0.77 ± 0.03	0.48 ± 0.03	0.34 ± 0.01	1.74 ± 0.11	0.70 ± 0.05	0.23 ± 0.01	0.18 ± 0.00	3.37 ± 0.30	0.78 ± 0.03
Evol-KMeans	0.43 ± 0.00	0.42 ± 0.00	0.93 ± 0.00	0.01 ± 0.00	0.92 ± 0.00	0.65 ± 0.00	0.49 ± 0.00	0.00 ± 0.00	0.65 ± 0.00	0.46 ± 0.00	0.84 ± 0.00	0.01 ± 0.00
**DynEC (Ours)**	**0.56 ± 0.06**	0.31 ± 0.01	1.10 ± 0.07	**0.04 ± 0.02**	0.85 ± 0.06	0.54 ± 0.05	0.62 ± 0.09	0.02 ± 0.02	**0.65 ± 0.06**	0.38 ± 0.02	0.96 ± 0.07	0.03 ± 0.01

Values are reported as Mean ± Standard Deviation over 5 independent runs. Best results are highlighted in bold. Paired *t*-tests over the five seeds show that the proposed method significantly improves ARI over K-Means and Evol-KMeans in City A (*p* = 0.0208 and *p* = 0.0107) and significantly reduces CSR relative to K-Means in all three cities (*p* < 10^−3^). The ARI differences against the strongest snapshot-oriented baselines in City B and City C are not statistically significant (*p*>0.05).

#### 4.4.1 Clustering quality (ARI & SC)

In City A (mixed type), which exhibits the highest volatility, the ARI (0.56 ± 0.06) of our DynEC method outperforms all other approaches, including spectral clustering (ARI 0.51 ± 0.01) and the explicit time-regularized baseline Evol-KMeans (ARI 0.43 ± 0.00). This indicates that DynEC is highly capable of identifying genuine behavioral semantics amid complex mixed patterns. However, static methods like K-Means show stronger intra-cluster geometric compactness (SC and DBI) on individual snapshots. This is an expected trade-off, as our evolutionary learning framework sacrifices minor instantaneous spatial cohesion to achieve a significantly lower Cluster Switching Rate (CSR), ensuring temporal semantic consistency. In City B (residential), stability-oriented baseline models (such as Evol-KMeans) achieved exceptionally high snapshot quality (ARI 0.92 ± 0.00, SC 0.65 ± 0.00), indicating that residential patterns form distinct and geometrically well-separated clusters on individual days. Although DynEC's ARI (0.85 ± 0.06) is slightly lower, it remains highly competitive while maintaining a consistent evolutionary representation. In City C (industrial), DynEC remains competitive compared to Evol-KMeans (ARI 0.65 ± 0.06 vs. 0.65 ± 0.00) and outperforms the state-of-the-art EvolveGCN (ARI 0.65 ± 0.06 vs. 0.62 ± 0.07), confirming its robustness across different consumer types.

##### 4.4.1.1 Temporal stability (CSR)

The proposed method achieves CSR values close to zero (0.02–0.04) across all cities, significantly lower than those of static baseline methods (K-Means/Spectral: approximately 0.70–0.79) and dynamic methods (EvolveGCN: approximately 0.29–0.61). However, Evol-KMeans further reduces CSR to 0.00–0.01 through explicit centroid smoothing; yet, its weaker ARI on the heterogeneous City A dataset indicates that temporal smoothing alone is insufficient to model mixed user drift. Although static methods such as K-Means may achieve high snapshot quality in stable environments (City B), their high CSR indicates frequent “identity switching,” where users are reassigned to different clusters daily due to minor fluctuations. Our evolutionary learning framework effectively smooths these transitions while maintaining semantic consistency, which is crucial for real-world utility applications.

##### 4.4.1.2 Statistical significance

Paired *t*-tests over the five random seeds confirm that DynEC's ARI improvement in City A is statistically significant relative to K-Means (*p* = 0.0208) and Evol-KMeans (*p* = 0.0107). For temporal stability, DynEC achieves significantly lower CSR than K-Means in City A, City B, and City C (all *p* < 10^−3^). By contrast, the ARI differences between DynEC and the strongest snapshot-oriented baselines in City B and City C are not statistically significant (*p*>0.05), which is consistent with the limitation discussed in Section 4.8.

### 4.5 Ablation study

To rigorously verify the contribution of the two core innovations of the proposed framework, a component-wise ablation study was conducted on the City A dataset. The results explicitly support the architectural design choices.

#### 4.5.1 Validation of multi-view dynamic graph construction

Regarding the removal of the time-aligned view (cDTW) (i.e., “w/o DTW View”), this operation resulted in the most significant decline in clustering quality, with ARI dropping sharply from 0.62 to 0.35. Therefore, geometric proximity alone is insufficient to characterize complex load patterns; the cDTW view is crucial for capturing shape similarity and identifying consistent user behavior in the presence of temporal misalignment. Similarly, removing the correlation view (Pearson) resulted in reduced stability (CSR increased from 0.02 to 0.05) and a slight decrease in ARI, confirming that statistical co-movement provides supplementary information and enhances the robustness of the clustering process.

#### 4.5.2 Validation of deep evolutionary graph learning framework

Setting the time-consistency weight to λ = 0 (“no time consistency”) resulted in a significant deterioration of the ARI (from 0.62 to 0.50), although the CSR remained at a low level. This indicates that without time regularization, the model cannot maintain a consistent semantic interpretation over time, even when cluster assignments do not fluctuate rapidly. Furthermore, removing the gating mechanism (“no gating”) resulted in a comprehensive decline in performance (ARI dropped to 0.51, CSR rose to 0.07), validating its role as a learnable filter that selectively aggregates information-rich spatial neighbors while suppressing noise, thereby balancing plasticity and stability.

### 4.6 Parameter sensitivity analysis

**Figure 2:**
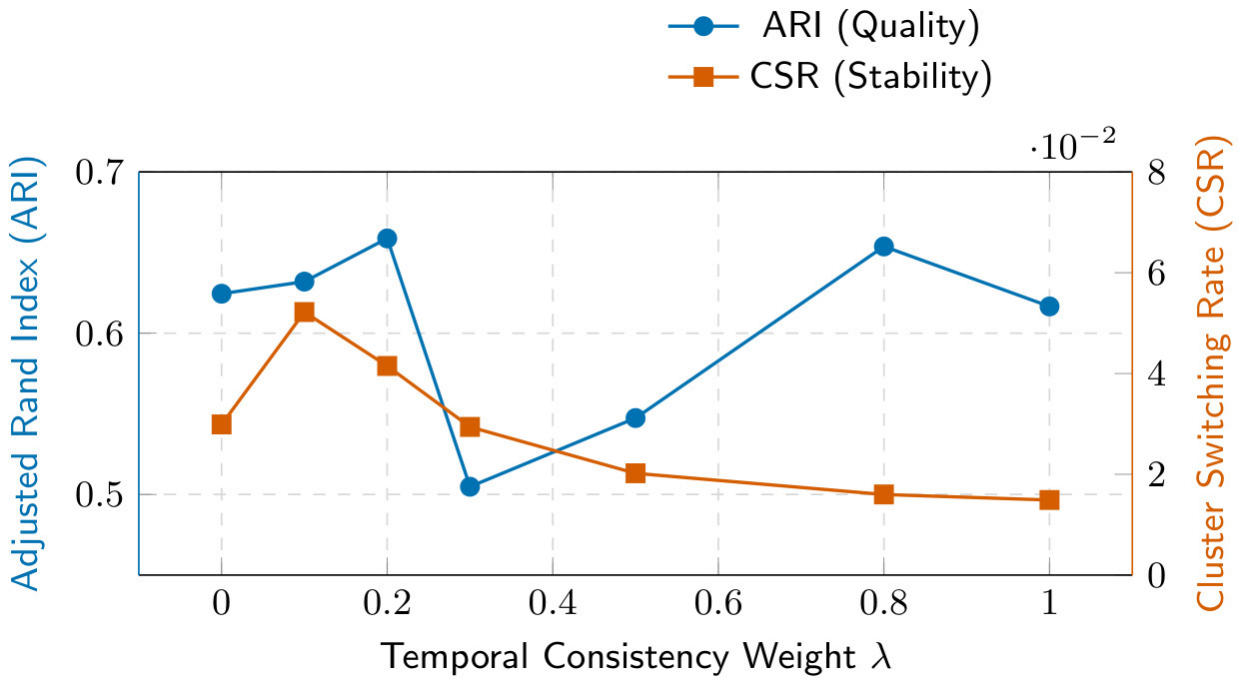
Sensitivity analysis of temporal consistency weight λ on City A. An optimal balance is observed around λ = 0.2, where cluster quality (high ARI) is maximized while maintaining temporal stability (low CSR).

**Figure 3:**
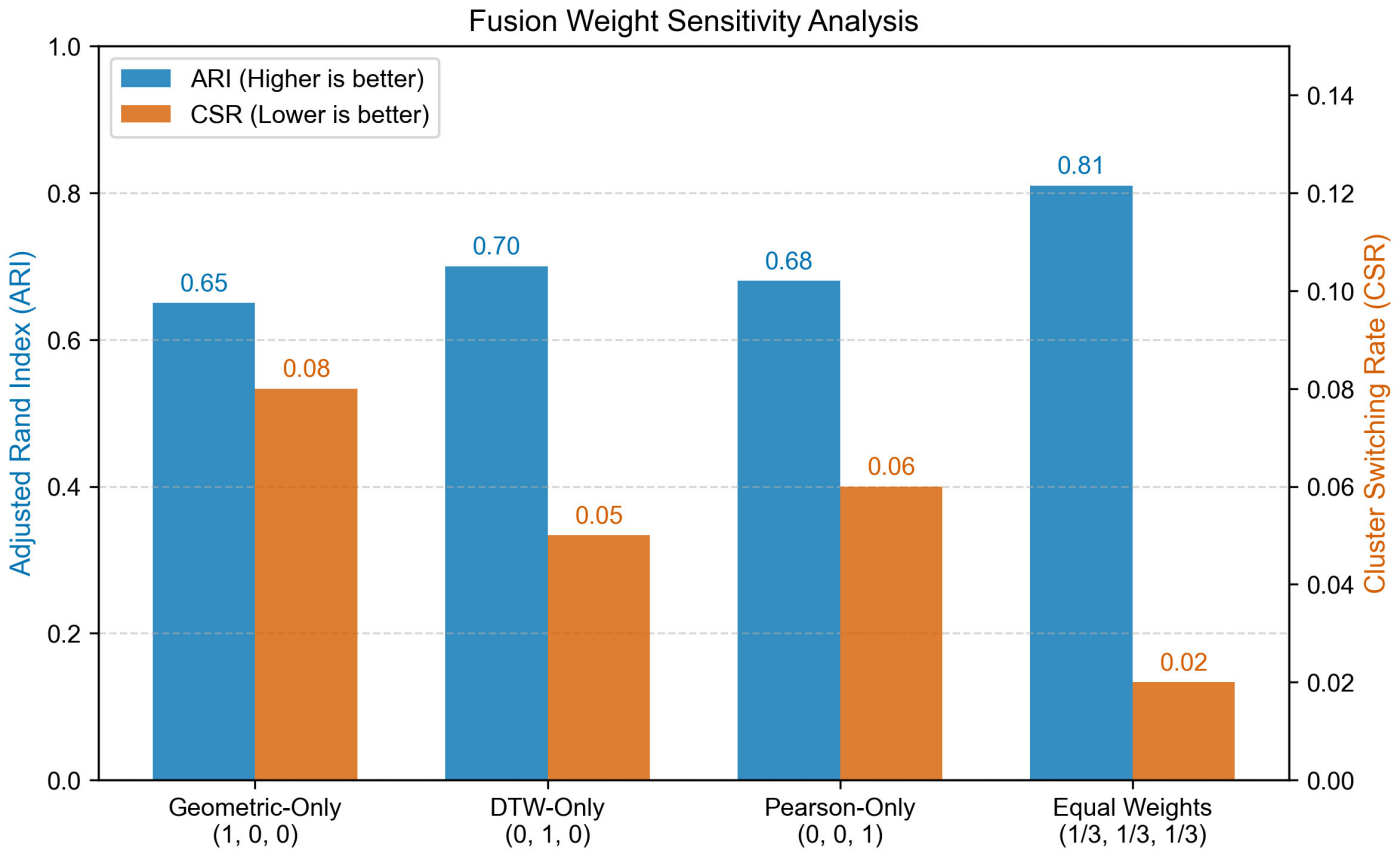
Sensitivity analysis of fusion weights. The equal-weight configuration (α = β = γ = 1/3) achieves the best trade-off, maximizing ARI while keeping CSR near zero.

As shown in Figure 2, the adjusted Rand index (ARI) and cluster switching rate (CSR) are plotted as λ varies from 0 to 1. In Mechanism I (λ < 0.1), the model lacks sufficient time constraints, resulting in poor cluster quality. Mechanism II (0.1 ≤ λ ≤ 0.3) represents the “optimal point,” where ARI peaks (at λ = 0.2) while CSR decreases, indicating that appropriate temporal regularization effectively filters out noise and improves alignment with the true values. In Mechanism III (λ > 0.5), although CSR decreases further (high stability), ARI fluctuates, suggesting that excessive regularization may hinder the model's ability to adapt to true conceptual drift, thereby leading to “lag.” In addition to λ, the model demonstrates robustness to the number of neighbors *k* in graph construction and the window size *w* in cDTW, as long as they capture sufficient local context (e.g., *k* ∈ [5, 15]). Figure 3 further shows that fixed equal fusion weights (α = β = γ = 1/3) provide the best balance between snapshot quality and temporal stability, supporting the engineering choice adopted in Section 4.2.

### 4.7 Case study: visualizing model effectiveness

To intuitively demonstrate the superiority of the proposed framework, qualitative visualizations corresponding to its two core innovations are provided.

#### 4.7.1 Robustness to phase shifts

**Figure 4:**
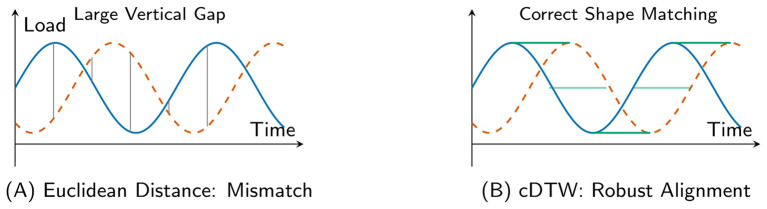
Illustration of robustness to phase shifts. Traditional Euclidean distance fails to match shape-similar but time-shifted load profiles, while the proposed cDTW view correctly aligns these profiles, ensuring they are clustered together despite the temporal misalignment. **(A)** Euclidean distance: mismatch. **(B)** cDTW: robust alignment.

The Multi-View Dynamic Graph Construction is designed to handle temporal misalignments. In the analysis of the mixed-type dataset, we identified numerous instances of “shape-similar but time-shifted” users (e.g., households with evening peaks occurring at 18:00 vs. 20:00). Methods based on traditional Euclidean distance (e.g., K-Means) often assign these users to disparate clusters due to large point-wise distances. However, by incorporating the cDTW view, the model successfully groups these users into the same functional cluster, confirming that the multi-view fusion strategy effectively captures inherent behavioral similarity beyond simple geometric alignment. As shown in Figure 4, cDTW correctly aligns shape-similar but time-shifted load profiles, whereas Euclidean distance treats them as mismatched due to point-wise temporal offsets.

#### 4.7.2 Stability under concept drift

**Figure 5:**
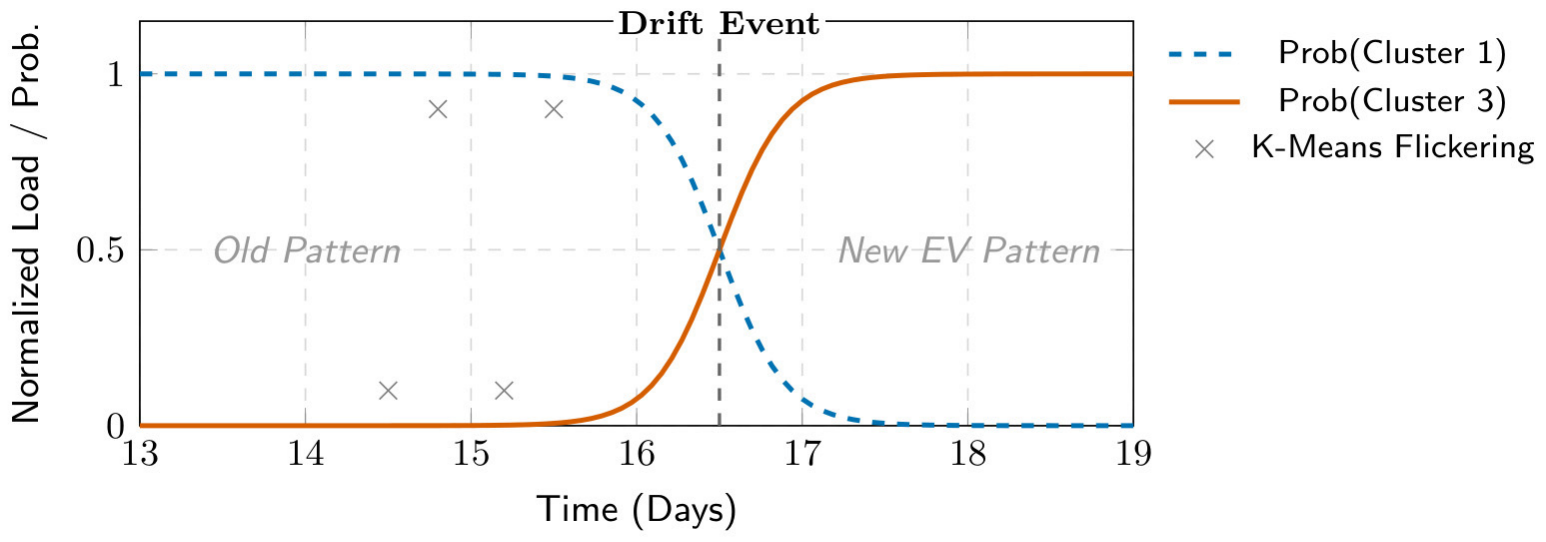
Visualization of concept drift for User #42. Top: Daily load profiles showing the emergence of V2G interaction patterns. Bottom: Cluster assignment probabilities over time. DynEC exhibits a smooth transition compared to the erratic switching of K-Means.

Figure 5 visualizes the evolution of User #42 from City A. This case exemplifies a typical scenario in the context of Source-Grid-Load-Storage: a residential user transforming into an active prosumer by participating in a Vehicle-to-Grid (V2G) program on Day 15. This structural change, characterized by the transition from passive consumption to bi-directional power flow, represents a distinct incremental concept drift.

As observed, the static K-Means algorithm, treating each day as an independent snapshot, reacted chaotically to the initial load fluctuations induced by V2G discharging events. On Days 14–16, the user's label flickered violently between Cluster 1 (Standard Residential) and Cluster 3 (V2G Prosumers). This phenomenon illustrates the “Identity Switching” problem induced by the stochasticity of source-load interactions, where static methods fail to distinguish between transient fluctuations and genuine behavioral evolution. Such instability would trigger erroneous billing adjustments in a real-world utility system.

In sharp contrast, DynEC maintained a coherent trajectory. Facilitated by the temporal consistency loss and the GRU memory mechanism, the model effectively suppressed immediate responses to transient noise. It reassigned the user to the V2G Prosumer cluster only after the new interaction pattern persisted and stabilized (post Day 16). This “smooth transition” capability confirms that our framework successfully balances plasticity (adapting to the new V2G mode) with stability (ignoring temporary volatility), validating its suitability for automated and noise-tolerant load profiling.

### 4.8 Discussion and limitations

While our method demonstrates exceptional performance in dynamic environments, we observe a slight performance trade-off in extremely stable settings. Specifically, in City B, where the load patterns are highly consistent, static K-Means marginally outperforms DynEC in terms of ARI on a single snapshot. This occurs because static models can greedily fit the cross-sectional data without the “historical burden" of temporal smoothing. This represents an inherent limitation of evolutionary clustering algorithms, where maintaining low CSR introduces a slight regularization penalty on snapshot-specific fit in purely static scenarios.

#### 4.8.1 Event-driven concept drift validation

**Figure 6:**
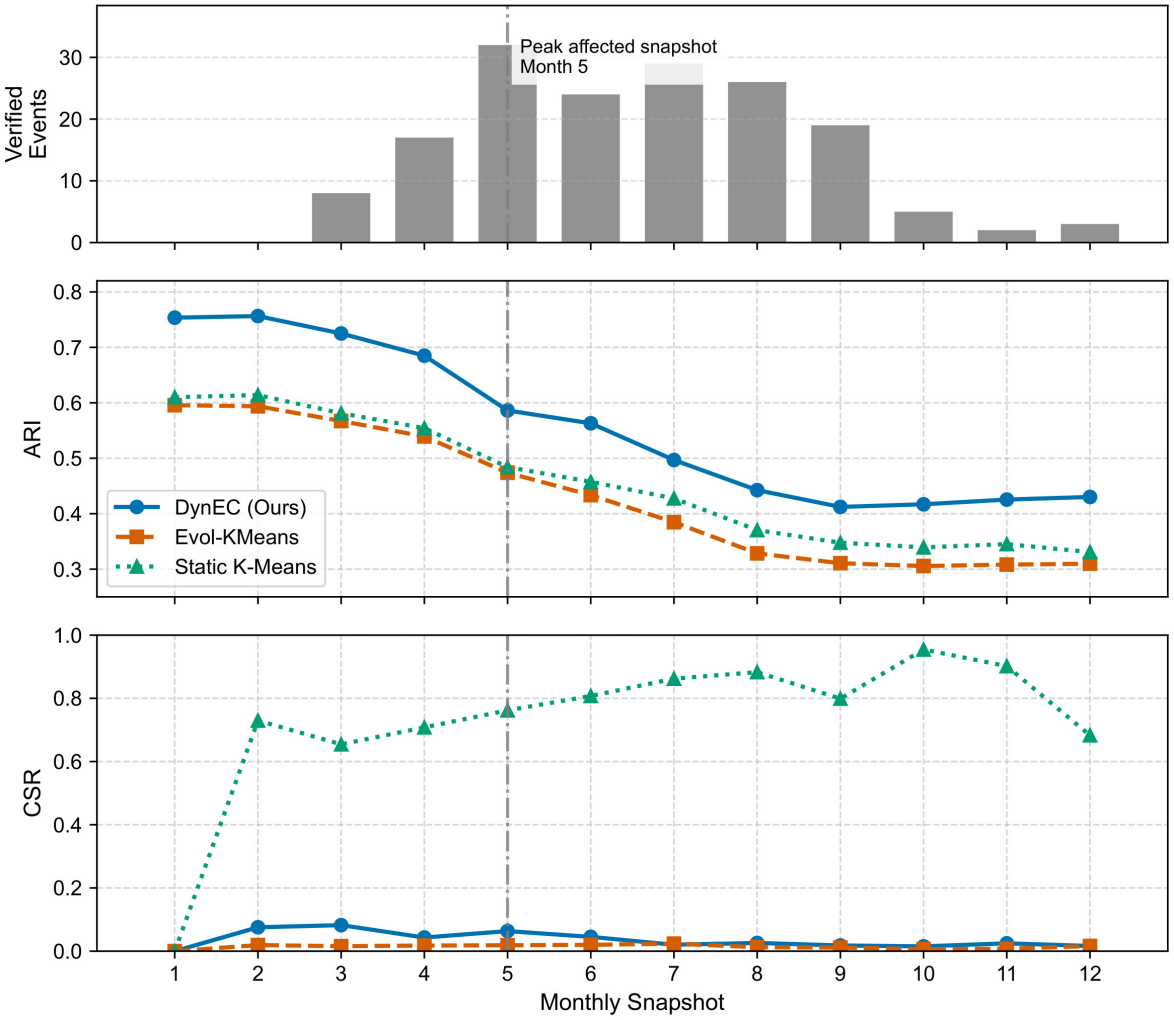
Event-driven concept drift validation based on the real City-A event log. **Top**: number of verified monthly events in the evaluated 500-user subset. **Middle and bottom**: mean ARI and CSR over five seeds. Month 5 contains the highest event concentration (32 users), where our approach retains higher clustering quality while adapting more selectively than static K-Means and less conservatively than Evolutionary K-Means.

To verify that the exceptionally low CSR achieved by DynEC is not merely the result of mathematical over-smoothing, we analyze its behavior around real-world events. Using the verified City-A event log for the same 500-user evaluation subset, we aggregate recorded EV, PV, and SHOCK events into monthly snapshots and compare them with the mean ARI/CSR trajectories over five seeds. As shown in Figure 6, Month 5 contains the highest concentration of verified events (32 affected users). Around this peak-drift snapshot, the proposed model preserves a clearly higher ARI than Evolutionary K-Means while allowing a moderate CSR increase, whereas Evolutionary K-Means keeps CSR near zero by over-smoothing, and its ARI drops steadily after the event accumulation period.

## 5 Conclusion

In this article, we presented the DynEC framework to address the challenges in Source-Grid-Load-Storage integration environments. By modeling load profiling as a continuous evolutionary process rather than a set of static labels, it effectively mitigates the identity-switching behavior caused by bi-directional source-load interactions. Our analysis indicates that traditional static methods fail to capture the evolutionary characteristics of energy consumption and exhibit operational instability. To solve these problems, we utilized a Multi-View Dynamic Graph Neural Network as the underlying architecture.

The core technical contributions of our framework are summarized as follows: First, it integrates geometric, temporal (cDTW), and statistical dependencies to capture complex non-Euclidean correlations, thereby comprehensively addressing the limitations of single-metric similarity in dynamic environments. In addition, it introduces a unified paradigm that combines a Gated Spatio-Temporal Graph Encoder with Dual-Objective Evolutionary Optimization. This mechanism allows the model to learn evolution-aware representations while explicitly balancing the trade-off between snapshot clustering quality and temporal smoothness.

Extensive experiments on three real-world datasets—comprising mixed residential/commercial, residential-dominant, and industrial park zones—confirm that DynEC significantly reduces the Cluster Switching Rate (CSR) compared to static baselines, while maintaining state-of-the-art clustering quality (Silhouette Coefficient). By shifting from state-based analysis to process-based evolution tracking, the proposed approach establishes a robust foundation for dynamic pricing and targeted demand response. Ultimately, DynEC strikes a critical balance between stability and adaptability, paving the way for the next generation of reliable and automated smart grid management.

## Data Availability

The Data availability statement is accurate. The datasets and code are available at: https://github.com/jerryao/DynEC.
